# Working Together May Be Better: Activation of Reward Centers during a Cooperative Maze Task

**DOI:** 10.1371/journal.pone.0030613

**Published:** 2012-02-15

**Authors:** Austen L. Krill, Steven M. Platek

**Affiliations:** 1 Georgia Gwinnett College, School of Liberal Arts, Lawrenceville, Georgia, United States of America; 2 University of Liverpool, School of Biological Sciences, Liverpool, United Kingdom; 3 University of Liverpool, Magnetic Resonance and Image Analysis Research Centre, Liverpool, United Kingdom; Cajal Institute - Consejo Superior de Investigaciones Científicas, Spain

## Abstract

Humans use theory of mind when predicting the thoughts and feelings and actions of others. There is accumulating evidence that cooperation with a computerized game correlates with a unique pattern of brain activation. To investigate the neural correlates of cooperation in real-time we conducted an fMRI hyperscanning study. We hypothesized that real-time cooperation to complete a maze task, using a blind-driving paradigm, would activate substrates implicated in theory of mind. We also hypothesized that cooperation would activate neural reward centers more than when participants completed the maze themselves. Of interest and in support of our hypothesis we found left caudate and putamen activation when participants worked together to complete the maze. This suggests that cooperation during task completion is inherently rewarding. This finding represents one of the first discoveries of a proximate neural mechanism for group based interactions in real-time, which indirectly supports the social brain hypothesis.

## Introduction

Cooperation is integral to facilitating survival [1]. Kin selection theory [2] describes why individuals and animals cooperate and behave altruistically toward genetically related individuals. However, the theory of kin selection does not fully explain why humans (and certain other species) have evolved to cooperate with individuals other than kin and often do so in ways where reciprocity is not direct or instantaneous. Both direct reciprocity, the opportunity to cooperate with another person when it is likely you will meet again, and indirect reciprocity, where the likelihood that you will meet again is very low, require complex computational systems of analysis to determine whether it is beneficial to partake in such cooperation [3].

The brain guides humans in realizing that accepting altruism without reciprocating is only beneficial in the short-term. Humans, because of theory of mind, also have the capacity to weigh long-term rewards, consequences, and circumstances resulting in decisions that allow for the survival of mutual cooperation [4]–[5] in the absence of genetic relatedness. Human theory of mind (ToM) allows for the representation of one's physical and mental states including motoric, mental, emotional, perceptual, and visceral [6] – self-awareness. This highly developed sense of self-awareness has allowed humans the unique capacities to think about themselves and to also consider the states of mind [7]–[9] and to make inferences about the mental states of others [10]–[11]. It is the cornerstone of the human ability to deceive, empathize, cooperate, and to interpret complex body language and a cornerstone of sociality [12]–[13].

Along with this unique ability to understand others and their motives, there is also a unique pattern of brain activation that accompanies cooperation with human partners. Rilling et al. [4] found that the caudate, rostral ACC, and OFC are linked to social cooperation; however activation in the ACC and caudate may be specifically linked to cooperation and human interaction as they were not evident with computer mediated cooperation.

The link between cooperation and theory of mind are essential in the current study because we hypothesized that ToM and the reward centers of the brain would be active when participants were tasked with working together, cooperating, to solve a motoric task – driving through a maze. Furthermore, it was hypothesized that in instances where participants reached the end of the maze while instructing their partner, activation in the reward network would be greater than the activation in the reward network when completing the maze alone in the self-drive condition [4].

## Methods

### Ethics Statement

The study was approved by the University of Liverpool, School of Biological Sciences Committee on Research Ethics. All participants provided written informed consent to participate in the study.

### Participants and Task

Twenty eight participants (14 pairs; M_age_ = 24.5; SD = 4.04; 18 females; 10 males) were recruited. Participants were asked to work together to complete a series of mazes while engaged in simultaneous fMRI scanning (hyperscanning). In this interactive task both subjects cooperated while undergoing simultaneous real-time fMRI to reach the end of a series of mazes. A form of “blind driving” was used which required the participants to rely upon one another and work together to reach the end of the maze. One participant served as the ‘instructor’ and the other as the ‘driver’. The instructor can view the maze field; however he cannot interact with the maze. In order to navigate the maze he must send directions, using the keypad, to his partner, the ‘driver’. The driver then receives the instructions, which appear in the form of numbers that are coordinated with directional instructions (left, right, forward, and backward) on an MRI-safe response pad. The driver presses the appropriate button on his response pad to move through the maze, but he is blind to the maze environment. This would be tantamount to getting into the driver seat of an automobile while wearing a blindfold and following instructions on what actions to take from the passenger (e.g., press accelerator, press brake, turn left, right, stop, etc). See (Figure S1).

Sixty seconds were allotted for the completion of each maze. Participants were informed that they would be working against the clock to find the end of the maze. Mazes were presented sequentially in pairs of 2 with a 20 second rest/scrambled image screen presented at the beginning, end, and in between mazes. All participants undertook the role of instructor for four mazes (instruct condition), completed 4 mazes on their own (self-drive condition), and finally undertook the role of driver (drive condition) for 4 mazes. Participants were randomly assigned a starting role as instructor or driver and participants completed the self-drive condition while the other participant received their structural scan to reduce overall time in the MRI environment. The order of mazes was randomized across participants.

### Imaging parameters

Participants were scanned using one of two scanners: Siemens Symphony 1.5 Tesla and a Trio 3 Tesla scanner. While the use of scanners that differ in field strength is not optimal, sequences for the two MRI scanners were optimized to make the imaging parameters as analogous as possible. In the Symphony scanner, functional images were collected using an EPI sequence (TR = 3000 ms, TE = 45 ms, FOV 192×192 mm, slice thickness = 3.5 mm, gap = .5, number of slices = 35). Participants also underwent a 7-minute structural scan (MPRAGE: 176 slices, TR = 2040 ms, TE = 3.93 ms). The remaining participants were scanned using a Trio 3T scanner (TR = 3000 ms, TE = 30 ms, FOV = 192×192 mm, slice thickness = 3.0 mm, gap = 3.3 or 10%, number of slices = 42). The parameters of the MPRAGE structural scan are the same as above except that the TE = 5.5 ms.

### fMRI Imaging Analysis

For pre-processing and statistical analysis of the fMRI data the researchers used the FMRIB Software Library. Single subject pre-processing was done for each participant, correcting for motion using MCFLIRT [14] and brain extraction using the BET tool [15]. Images were also intensity normalized and smoothed (full width half max = 6). All higher-level analyses were performed using fMRI Expert Analysis Tool (FEAT) version 5.98 [15] and mixed effects modeling. After the pre-processing, first level contrasts for each condition were created: drive, self-drive, instruct. They were then entered into higher-level mixed effects analyses to get the combined results from both scanners and participants: collapse drive (drive condition 1.5T+drive condition 3T), collapse instruct (instruct condition 1.5T+instruct condition 3T), collapse self-drive (self-drive condition 1.5T+self-drive condition 3T), collapse instruct versus collapse drive (collapse instruct−collapse drive), collapse self-drive versus collapse instruct (collapse self-drive−collapse instruct), collapse self-drive versus collapse drive (collapse self−collapse drive). Finally contrasts were combined for the pair (drive+instruct conditions) versus self-drive contrast ([collapse drive+collapse instruct]−[collapse self-drive]) and the self-drive versus pair contrast ([collapse self-drive]−[collapse drive+collapse instruct]) to show the activation when one participant is instructing and the other is driving and they are working together to solve the maze. All higher level contrasts were set to a z = 2.3, p<0.05, unless otherwise stated. These contrasts allowed us to examine patterns in activation when participants were working together versus working alone in the maze task.

In the second part of the analysis, the same pre-processing and statistical analyses were performed in the same way as discussed previously. After pre-processing we created first level contrasts at the individual level for all of the instruct and self-drive conditions. The drive conditions, except for self-drive, were not included in the contrasts because participants were not given feedback about whether or not the maze was completed; therefore, they did not know if they had successfully completed the maze or not. In the second level analysis, conditions were contrasted at the individual level. For example if a participant completed the maze during the self-drive condition in the first part of the round, but did not complete it in the second part of the round (each maze set has 4 mazes- 2 pairs of 2 mazes), and then completed both rounds of the mazes during the instruct condition then his contrast would be something like this: self-drive complete – self-drive not complete; self-drive complete – instruct complete; self-drive not complete – self-drive complete; self-drive not complete – instruct complete; instruct complete – self-drive not complete; instruct complete – self-drive complete. In other words, each participant's condition was contrasted at the individual level with the other possible outcomes.

Third-level analysis brought everything to the group level. Contrast Parameter Estimate (COPE) files were combined for all participants that fell into each category. Using the previous example, that participant's COPE files would have been combined with other participants' for each contrast, so all participants COPE files were combined under the proper category to create group activation means. FMRI data processing was carried out using FEAT (FMRI Expert Analysis Tool) version 5.98, part of FSL (FMRIB's Software Library, www.fmrib.ox.ac.uk/fsl). Z (Gaussianised T/F) statistic images were thresholded at p = .05 (uncorrected). Images were thresholded (z>1.6) using non corrected significance threshold of p<.05 unless otherwise stated.

## Results

First level Instruct contrasts were collapsed across both scanners. Activation was evident in the following areas: bilateral frontal pole, bilateral medial frontal gyrus, right precentral and postcentral gyrus, right subparietal lobe and right inferior temporal gyrus. Furthermore, there was activation in areas that have been implicated in the ToM network: the precuneus, left anterior cingulate gyrus (ACC), the left superior temporal gyrus, and bilateral medial temporal gyrus (Table S1 for coordinates) Next self-drive contrasts were collapsed across both scanners. The following areas showed significant levels of activation during this task: bilateral orbitofrontal cortex, bilateral precentral and postcentral gyrus, bilateral posterior middle temporal gyrus, right paracingulate gyrus, and left precuneus cortex (See Table S2 for coordinates). No voxels survived cluster correction in the collapsed drive condition. The collapsed instruct versus collapsed self-drive contrast reveals activation that survived after all activation from the instruct condition was combined and then the collapsed activation from the self-conditions was subtracted (collapse instruct – collapse self). This contrast illustrates which areas were more active in the instruct conditions (e.g. what sets them apart from the self-drive condition). The areas where activation was seen are as follows: occipital pole, left precuneus, left inferior temporal gyrus, and right lateral occipital cortex. No activation survived the collapsed self-drive versus the collapsed instruct contrast. The collapse self-drive versus collapse drive contrast revealed activation in the left inferior frontal gyrus, precuneus cortex and posterior cingulate gyrus, right paracingulate gyrus, and left middle temporal gyrus. The contrast, pair versus self-drive, combined the driving and instruct conditions and contrasted them against the self-driving condition ([collapse drive+collapse instruct] – self-drive). Significant activation was evident in the precuneus cortex, orbital frontal cortex, postcentral gyrus, supramarginal gyrus (anterior and posterior division), left caudate and putamen, as well as the lateral occipital cortex. Finally in the self-drive versus pair contrast, the self-drive condition was contrasted with the paired driving and instruct conditions (self-drive−[drive+instruct]). Activation was found in the inferior frontal gyrus, precuneus, posterior cingulate gyrus, paracingulate, and middle frontal gyrus (See Table 1.)

**Table 1:** Pair versus Self-Drive Contrast.

Pair Activation	Hemisphere	X	Y	Z	z-score
Precuneus Cortex	L	−8	−56	62	4.28
Superior Frontal Gyrus	L	−22	4	−12	4.88
Postcentral Gyrus	L	−28	−40	44	3.91
Supramarginal Gyrus (anterior and posterior)	L	−28	−40	44	3.91
Superior Parietal Lobule	L	−12	−60	62	3.68
Putamen	L	−22	2	−6	4.23
Caudate	L	−16	18	0	3.88
Lateral Occipital Cortex	R	34	−88	6	3.48
Occipital Pole	R	36	−88	10	3.36
**Self Activation**					
Inferior Frontal Gyrus	L	−46	16	18	4.86
Precuneus	L	−2	−58	42	4.68
Superior Frontal Gyrus	L/R	0	20	52	4.02
Paracingulate Gyrus	R	6	24	54	3.84
Middle Frontal Gyrus	L	−44	10	34	4.68
Posterior Cingulate Gyrus	R	12	−52	34	4.04

Activation coordinates and z-scores from *Pair versus Self-Drive* (top) and *Self-drive versus Pair* (bottom).

In this second portion of the analysis brain activation between those who completed the maze and those who did not complete the maze was compared. Ultimately, comparisons were made between these four conditions: Instruct Complete (IC), Self Complete (SC), Instruct Did Not Complete (IDN), and Self Did Not Complete (SDN) (See Table 2).

**Table 2:** Conditions and descriptions for comparison between completed and incomplete maze attempts.

Condition	Description
Instruct Complete (IC)	The maze was completed during the instruct condition.
Self Complete (SC)	The maze was not completed during the self-drive condition.
Instruct Did Not Complete (IDN)	The maze was not completed during the instruct condition.
Self Did Not Complete (SDN)	The maze was not completed during the self-drive condition.

Comparisons were made between all possible permutations of these four conditions (See Table S3). The contrast, IC –IDN, compared those who completed the maze during the instruct condition versus those who did not complete the maze when they were instructing. Activation was evident in the following areas of the brain: left caudate, bilateral temporal lobes, left posterior cingulate gyrus, and left putamen. In the contrast, IC-SC, activation was contrasted between participants who finished the maze during the instruct condition and participants who completed the maze during the self-drive condition. Activation appeared in the following substrates: left precentral gyrus, right precuneus, cerebellum, left superior frontal gyrus, right lingual gyrus, right postcentral gyrus, left post central gyrus, left precentral gyrus. Activation was evident in the following substrates for the SC - SDN contrast: Bilateral paracingulate gyrus, right anterior cingulate gyrus, left posterior cingulate gyrus, bilateral precuneus cortex, bilateral middle frontal gyrus, left precentral gyrus, bilateral inferior frontal gyrus, bilateral frontal pole, left supramarginal gyrus. Finally in the SC-IC contrast, activation was evident in the left anterior cingulate gyrus, bilateral frontal pole, caudate, paracingulate gyrus, bilateral superior temporal gyrus (posterior division), bilateral middle temporal gyrus, and the precuneus cortex.

## Discussion

Participants showed activation in putative ToM substrates (e.g. precuneus cortex, OFC, supramarginal gyrus, paracingulate gyrus) and reward centers (e.g. caudate and putamen) when cooperating with others. The activation patterns for the collapsed instruct condition (occipital pole, left precuneus, left inferior temporal gyrus, and right lateral occipital gyrus) suggest that participants were recruiting parts of the ToM network to process information as they gave instructions. In fact, substrates associated with the ToM network were evident in all of the contrasts: instruct, self-drive, pair versus self-drive, and self-drive versus pair. Because the task required participants to think about their own behavior in all conditions, as well as to consider the actions of others in response to their instruction, the ToM network activation makes sense. Even in the conditions when the person was working alone, many of the same areas that are recruited for thinking about others may be recruited [7]–[8], which is consistent with Gallup's hypothesis [7].

Precuneus activation was present in all conditions. Activation in the precuneus has been associated with mentalizing about cooperation, the self and others [16]–[17]. In stories describing deception, cooperation, or combined cooperation and deception the precuneus seems to be particularly involved in processing information pertaining to cooperation [18]. In part, the findings correspond to this suggestion, as the instructor was working to cooperate with the driver on the maze task, activation was evident in the precuneus. Furthermore, left precuneus is one of the regions activated when attributing emotions to other people and the self [18]–[19]. Additionally, the precuneus has also been connected to visuo-spatial imagery. The precuneus is linked to motor imagery and abstract mental imagery tasks [20]. Because of the nature of the maze task, some of the precuneus activation is likely related to the visuo -spatial imagery components of the maze task.

Furthermore, activation in the superior and medial frontal gyri was evident in all of the conditions. Activation has been reported in the superior frontal gyrus and the medial frontal gyrus in response to cooperation stories [18]. Activation in these areas has been related to both cooperation and deception, but there were some differences in the degree of activation given the type of story (e.g. cooperative or deceptive) [18]. Portions of the superior frontal gyrus and the medial frontal gyrus are also considered part of the medial prefrontal cortex (with connections to the OFC) and are consistently reported in ToM tasks [21]–[24], [18]. Research has shown that the medial prefrontal cortex and the ACC are implicated in making the distinction between self and other [18], [25], [9]. During the instruct condition one could interpret these findings as suggesting that participants were thinking about what they were doing and what needed to be communicated to their partner-participant to facilitate movement through the maze.

### Overlap in Neural Activation on Self-Drive and Instruct Conditions

**Figure 1:**
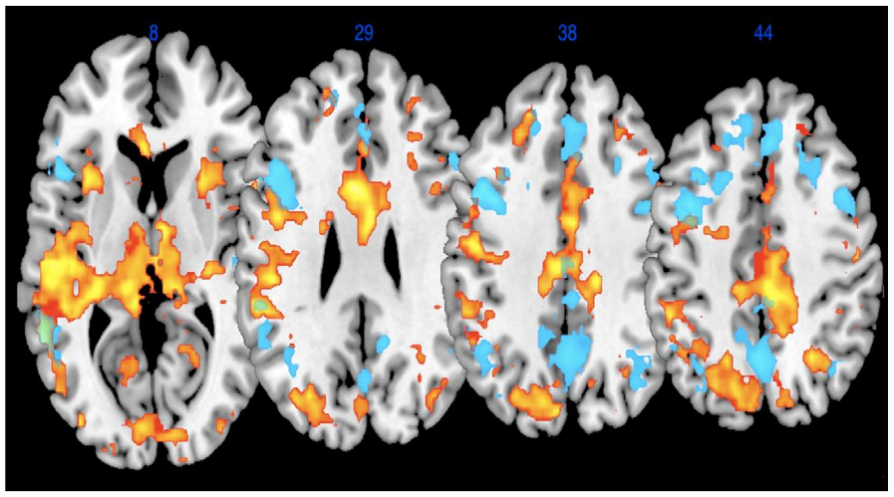
Collapse Self-Drive and Collapse Instruct. Overlay of Collapse Self-Drive (yellow) and Collapse Instruct (blue). Figures are in neurological orientation.

The self-drive condition revealed activation in many of the same substrates that were active in the instruct condition (See Figure 1). An overlap in the brain areas associated with working alone to complete a task and cooperating with another person emerged in this condition; that is, participants likely simulated one's self in that situation and used that to model the mental state of another [7]–[9]. Many of the same areas recruited for self-reflection are also recruited for ToM [18]. Johnson et al. [26] found anterior medial prefrontal activation and posterior cingulate activation in their examination of self-reflection. A similar situation arose in this study. Participants in the self-drive condition showed activation that overlapped with the pair drive and instruct activation, suggesting that the active substrates may not be specifically self-reflective ToM functions, but that they are involved in metacognitve functioning, generally.

### Executive Function and Reward

The collapsed self-drive condition revealed activation in areas associated with executive function and reward. The lateral orbital frontal cortex (OFC) showed bilateral activation. In an attempt to tease apart the functions of the OFC, it was hypothesized that the lateral OFC is heavily involved in the decision making process in situations that are “incompletely specified” and in suppressing previously rewarded responses ([27], p. 310). Circumstances requiring individuals to make judgments with an incomplete set of information creates an element of unpredictability, and this appears to activate areas of the lateral OFC [27]. Furthermore, research supports that the OFC monitors reward values, and in a novel (or uncertain) situation an appraisal can be made resulting in appropriate response evaluation and selection [27]. Additionally it was reported that lateral OFC and caudate activation was evident in instances of short-term reward prediction [28]. In accordance with others, Tanaka et al. [29] reported that as immediate rewards were dispensed for learned actions, activity was evident in the lateral oribitofrontal cortex and the striatum.

These findings provide support for the role that the OFC may have played in the present study. Participants were unsure of the outcome of the task and the OFC may have been integral in assessing the task and making decisions about how to proceed. Additionally, the OFC may have also been activated upon the immediate anticipation and receipt of the reward, which was the completion of the maze. In particular the OFC may have been linked to the reward system in the pair versus self-drive contrast, as the caudate nucleus and putamen, areas that have been implicated in reward [30] were also active. It is our interpretation that where other components of the reward network are activated the OFC activation is possibly linked to reward activation.

### Reward Center Activation

**Figure 2:**
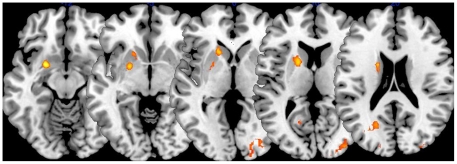
Activation in the caudate in the Pair versus Self-Drive Condition.

In the pair versus self-drive contrast activation was evident in three areas associated with reward: OFC, caudate, and putamen (Figure 2). The caudate and nucleus accumbens are known for their reward response [30], and they receive dopamine projections from the midbrain [4], [30]. It appears that the OFC is essential in the control of goal directed behavior [31]–[33]. Lesions of the OFC lead to impaired decision-making regarding the outcome of actions [34]. Because rewards are imperative to the primary goals of behavior, human motivation may be linked to the processing of reward stimuli in the OFC [35]. In other words, positive reinforcement is one of the primary functions of rewards, and the OFC is integral in this process.

### Reward Centers and Maze Completion

Substrates of the reward system in the brain were activated, specifically the OFC/MPFC, caudate and putamen when the IC activation was contrasted with IDN and the SC was contrasted with IC conditions. Additionally reward network activation survived several of the other contrasts, and this activation appears to be linked to completing the maze by oneself or during the cooperation condition, but the main component is that the reward areas are active during successful maze completion.

A higher level contrast was run between (IC – IDN) – (SC – SDN) and (SC – SDN) – (IC –IDN) to investigate the unique brain activation between instruct complete and self complete conditions, controlling for activation in the instruct did not complete (IDN) and the self did not complete (SDN) conditions. Results indicated that the caudate and putamen were significantly more active (p<.05) in the (IC – IDN) – (SC – SDN) contrast. This suggests that in the incidents where the participants were working together *and* successfully completed the maze activation in the putamen and caudate, reward centers of the brain, was significantly stronger than in the conditions where participants completed the maze alone. According to Damasio the caudate and putamen serve the following functions: “The putamen is involved mainly in evaluating actions in terms of sensory contexts and rewards, whereas the caudate nucleus is involved mainly in comparing actual and predicted rewards for learning” ([36] p. 948). Putamen and caudate activation are also related to anticipation of reward [28]. Furthermore, this finding appears to provide support for our previous conclusion that the caudate, and perhaps the putamen as well, may be uniquely related to the reward that accompanies engaging in successful cooperation to achieve a goal with another individual or the anticipation of such a reward.

### Conclusion

One goal of this study was to examine how cooperation (two individuals working together on a task simultaneously) differed from doing a task alone. The control task, or self-drive condition was designed to provide a basis of comparison for activation when one is working alone. While there was a great deal of overlap in the self-drive condition and the instruct condition, there was a distinct difference in the pair (or combined brain vs. the self-drive). Reward centers in the brain were active in the pair contrast. Thus, it appears that variation in the reward system is activated depending upon whether one completes the maze alone or in cooperation with another person. This activation difference might suggest that the participants experienced some anticipation about the successful completion of the maze via communication with their partner. While it is rewarding to navigate the maze by oneself, as evidenced by activation in the OFC, it may be more rewarding to navigate the maze under conditions of real time cooperation. Activation in the OFC was evident in this contrast and most importantly caudate and putamen activation was present. The activation in the caudate and putamen is unique to this contrast, and these parts of the brain seem to play a special role in cooperation and reward [37]. Parts of the reward system network were dissociated, and it was found that anticipation of reward resulted in activation in the caudate and the putamen, whereas notification that the reward was earned revealed activation in the medial prefrontal cortex [28]. Others report that the caudate is associated with cooperation when one is making decisions about social reward for their partners during a game [30]. Activation in the caudate and anterior cingulate gyrus was found following cooperation in a Prisoner's dilemma game [10]. Specifically, this response is isolated to the condition where participants were cooperating with another human being. Perhaps the caudate and putamen are particularly sensitive to human interaction and the rewards associated with it. As Rilling suggests [10] the incorporation of the reward system in cooperative interactions has helped to lay the groundwork and maintain human cooperation among individuals who are not kin.

This study has shown that the theory of mind network is associated with cooperation in this task as well as when receiving and considering instructional requests/needs of a cooperator. Additionally, many of the same neural correlates associated with cooperation are also incorporated in self-reflection, as evidenced by the activation patterns in the self-drive condition. Most importantly, activation in the caudate nucleus and putamen was apparent only when the participant was cooperating and working with the other participant, in the combined brain contrast. In the second part of the study results indicated that in fact, caudate and putamen were the unique remaining activations in the critical contrast between the instruct complete condition and the self complete conditions: (IC – IDN) – (SC – SDN). Components of the reward system were activated when completing the maze with their partner. This unique activation may suggest that there is a particular part of the reward system, possibly residing in the caudate or putamen, that is involved in the reward associated with human (i.e. conspecific) social interaction. However, caudate and putamen activation was still evident in some of the self-complete contrasts as well as the other IC conditions. So the reward center activation is not completely limited to working with another person.

There are several important limitations in this study that should be addressed. First, we did not do our analysis on an individual by individual basis. The contrasts were collapsed across all subjects so one should exercise caution in making generalizations and assuming that this activation pattern was evident for all subjects. There are definitely individual differences to take into account, but they are not examined in this analysis. We can say that on an “average” it appears that these areas of the brain were more active and involved with the completion of the maze with another person. Future investigations that carefully measure individual variation on traits such as, but not limited to personality, social ability, or mindfulness would add greatly to our understanding of this socialization based reward process. Secondly, we did not collect precise temporal information for this task. We did not account for the time it took to complete the maze in our analysis because all of the mazes were randomly assigned. Therefore, the issue of task difficulty comes into question. If the task was more difficult, as one assumes that it might be when the participant was trying to instruct another person, then it is fair to say that it is possible that some of the reward center activation could be related to task difficulty rather than cooperation. We did however, vary maze difficulty with some of the mazes being very easy and other being more challenging which were randomly assigned to participants and should have mitigated some of the possible effects of task difficulty.

Overall, this study demonstrated that working together to complete the maze resulted in the greatest activation in the caudate and putamen reward areas, compared to other conditions. This finding provides a proximate neural underpinning for Dunbar's social brain hypothesis [13].

## Supporting Information

Figure S1 **Visual display of maze environment.**
(DOCX)

Table S1 **Instruct Condition coordinates and activation.**
(DOCX)

Table S2 **Self-Drive condition coordinates and activation.**
(DOCX)

Table S3 **Complete versus Incomplete by Instruct and Self-drive conditions activations.**
(DOCX)
