# Supplementary figures and images for: Working Together May Be Better: Activation of Reward Centers during a Cooperative Maze Task
Source: PLoS One. 2012 Feb 15;7(2):e30613. doi: 10.1371/journal.pone.0030613 (PMC3280262; doi:10.1371/journal.pone.0030613)

**Supporting Information**

**Figure S1. Visual display of maze environment**


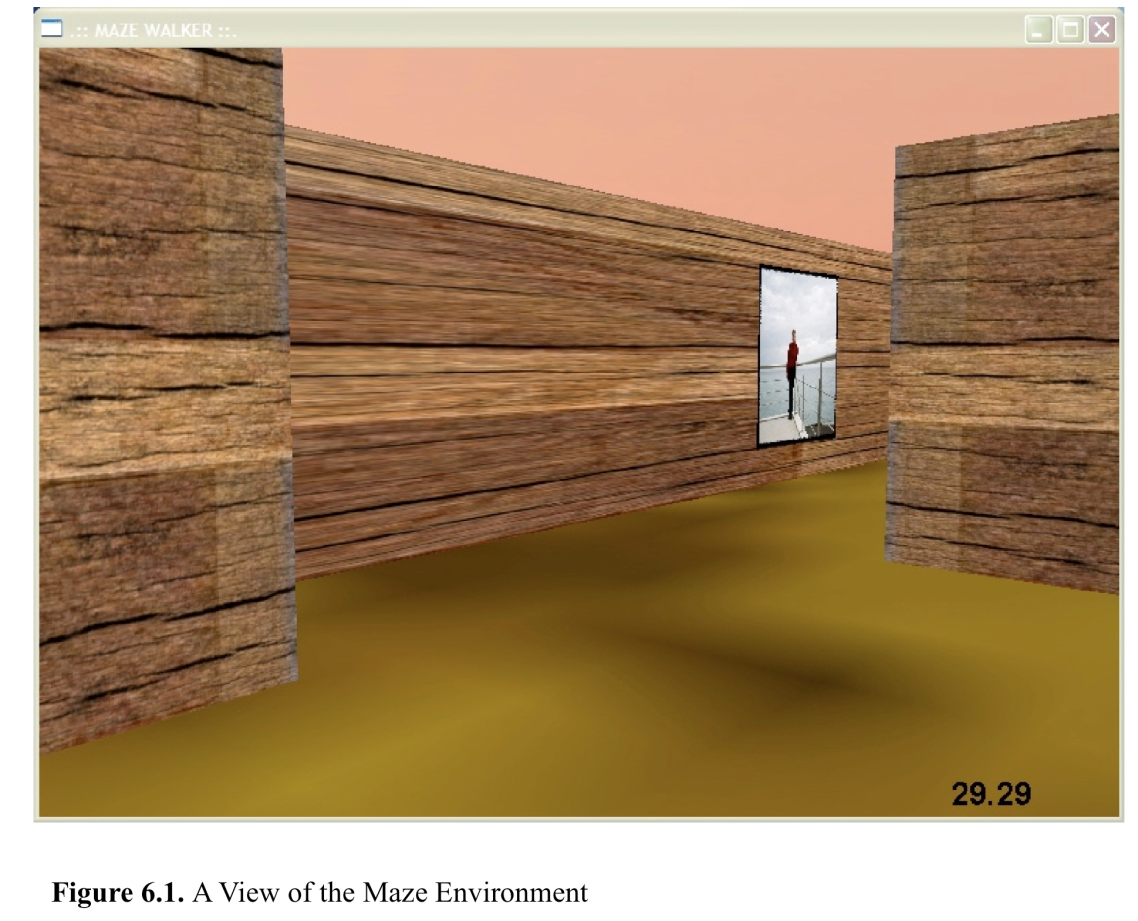

Supplement: Figure S1 — Visual display of maze environment. (DOCX) [file pone.0030613.s001.docx]
