# Supplementary material for: Working Together May Be Better: Activation of Reward Centers during a Cooperative Maze Task
Source: PLoS One. 2012 Feb 15;7(2):e30613. doi: 10.1371/journal.pone.0030613 (PMC3280262; doi:10.1371/journal.pone.0030613)
Supplement: Table S1 — Instruct Condition coordinates and activation. (DOCX) [file pone.0030613.s002.docx]

**Table S1. Instruct Condition coordinates and activation.**

| Anatomical Region | Right/Left | *X* | *Y* | *Z* | Z-score |
| --- | --- | --- | --- | --- | --- |
| Frontal Pole | R | -22 | 42 | 24 | 4.94 |
| Frontal Pole | L | -20 | 40 | 38 | 4.66 |
| Precuneus cortex | L | -4 | -40 | 68 | 5.98 |
| Superior temporal gyrus | L | -56 | -24 | 4 | 5.74 |
| Medial frontal gyrus | R | 22 | 36 | 22 | 4.42 |
| Medial frontal gyrus | L | -26 | 32 | 36 | 4.51 |
| Anterior cingulate cortex | L | -4 | 10 | 28 | 5.75 |
| Inferior temporal gyrus | R | 52 | -54 | -8 | 3.75 |
| Precentral gyrus | R | 50 | 8 | 40 | 4.4 |
| Postcentral gyrus | R | 6 |  | 68 | 6.11 |
