# Supplementary material for: Working Together May Be Better: Activation of Reward Centers during a Cooperative Maze Task
Source: PLoS One. 2012 Feb 15;7(2):e30613. doi: 10.1371/journal.pone.0030613 (PMC3280262; doi:10.1371/journal.pone.0030613)
Supplement: Table S2 — Self-Drive condition coordinates and activation. (DOCX) [file pone.0030613.s003.docx]

**Table S2. Self-Drive condition coordinates and activation**

| Anatomical Region | Right/Left | *X* | Y | *Z* | z-score |
| --- | --- | --- | --- | --- | --- |
| Postcentral gyrus | R | 54 | -56 | 26 | 4.24 |
| Orbitofrontal cortex | R | 42 | 24 | -6 | 5.16 |
| Oribitofrontal cortex | L | -40 | 32 | -8 | 5.13 |
| Lateral occipital cortex | R | 36 | -70 | 40 | 4.19 |
| Medial temporal gyrus (posterior) | R | 62 | -36 | -2 | 5.46 |
| Medial temporal gyrus | L | -56 | -52 | 6 | 5.9 |
| Paracingulate gyrus | R | 4 | 28 | 44 | 5.12 |
| Precuneus Cortex | L | -]4 | -40 | 68 | 5.98 |
