# Supplementary material for: Working Together May Be Better: Activation of Reward Centers during a Cooperative Maze Task
Source: PLoS One. 2012 Feb 15;7(2):e30613. doi: 10.1371/journal.pone.0030613 (PMC3280262; doi:10.1371/journal.pone.0030613)
Supplement: Table S3 — Complete versus Incomplete by Instruct and Self-drive conditions activations. (DOCX) [file pone.0030613.s004.docx]

**Table S3** **Complete versus Incomplete by Instruct and Self-drive conditions activations**

|  | **Hemisphere** | **X** | **Y** | **Z** | **Z-score** |
| --- | --- | --- | --- | --- | --- |
| IC-SDN |  |  |  |  |  |
| Frontal lobes | L | -16 | 16 | 50 | 2.93 |
| Precentral gyrus | L | -38 | -22 | 50 | 2.30 |
| Precuneus | L | -2 | -60 | 40 | 1.93 |
| Paracingulate | L | -6 | 20 | 46 | 2.62 |
| Superior frontal gyrus | L | -24 | 16 | 46 | 1.98 |
| Middle frontal gyrus | L | -40 | 22 | 30 | 2.85 |
| **IC-IDN** |  |  |  |  |  |
| Temporal occipital fusiform gyrus | L | -32 | -50 | -20 | 1.80 |
| Temporal occipital fusiform gyrus | R | 28 | -44 | -18 | 2.07 |
| Caudate | L | -18 | -26 | 22 | 2.00 |
| Posterior cingulate gyrus | L | -4 | -24 | 28 | 1.83 |
| Putamen | L | -26 | 16 | 4 | 2.33 |
| Juxtapositional lobule cortex | L | -14 | -16 | 44 | 2.01 |
| Juxtapositional lobule cortex | R | 12 | -8 | 50 | 2.13 |
| Anterior cingulate cortex | R | 12 | 4 | 36 | 1.93 |
| Superior frontal gyrus | L | -28 | -12 | 62 | 1.68 |
| Precentral gyrus | L | -30 | 0 | 32 | 2.43 |
| **IDN – IC** |  |  |  |  |  |
| Middle frontal gyrus | L | -24 | 2 | 52 | 2.25 |
| Middle frontal gyrus | R | 26 | 2 | 52 | 1.71 |
| Precentral gyrus | R | 26 | -10 | 44 | 2.38 |
| Paracingulate gyrus | L | -2 | 20 | 44 | 1.96 |
| Supramarginal gyrus | R | 44 | -44 | 44 | 2.68 |
| Anterior cingulate gyrus | L | -4 | 6 | 30 | 1.64 |
| Anterior cingulate gyrus | R | 2 | 0 | 32 | 2.79 |
| Superior frontal gyrus | R | 24 | 2 | 48 | 1.81 |
| Superior frontal gyrus | L | -22 | 2 | 56 | 2.23 |
| Precuneus | L | -4 | -52 | 52 | 1.62 |
| **IC – SC** |  |  |  |  |  |
| Precentral gyrus | L | -34 | -22 | 50 | 2.16 |
| Precuneus | R | 14 | -50 | 62 | 1.62 |
| Cerebellum | R | 26 | -50 | -32 | 2.43 |
| Superior frontal gyrus | L | -18 | 0 | 62 | 1.71 |
| Lingual gyrus | R | 22 | -40 | -8 |  |
| Supramarginal gyrus | L | -22 | -40 | 38 | 2.49 |
| Postcentral gyrus | L | 36 | -28 | 50 | 2.07 |
| Superior parietal lobule | L | -22 | -52 | 50 | 2.63 |
| **SC – IC** |  |  |  |  |  |
| Anterior cingulate gyrus | L | -2 | 36 | 16 | 2.49 |
| Frontal pole | L | -20 | 52 | 16 | 3.81 |
| Frontal pole | R | 22 | 54 | 16 | 3.48 |
| Caudate | L | -14 | 10 | 16 | 3.01 |
| Caudate | R | 16 | 8 | 16 | 2.24 |
| Paracingulate | L | -2 | 48 | 18 | 2.87 |
| Superior temporal gyrus (posterior) | L | -60 | -34 | 4 | 2.97 |
| Superior temporal gyrus (posterior) | R | 58 | -34 | 2 | 3.97 |
| Middle temporal gyrus | R | 62 | -32 | -4 | 3.087 |
| Middle temporal gyrus | L | -60 | -32 | -2 | 2.33 |
| Precuneus | L | -2 | -38 | 52 | 3.06 |
| **IDN – SDN** |  |  |  |  |  |
| Precuneus/post cingulate gyrus | R | 8 | -52 | 32 | 2.04 |
| Cingulate gyrus | L | -4 | -48 | 16 | 2.07 |
| Cingulate gyrus | R | 2 | -38 | 18 | 1.72 |
| Superior frontal gyrus | L | -20 | 38 | 46 | 2.22 |
| Middle temporal gyrus | R | 58 | -14 | -22 | 1.86 |
| Anterior cingulate gyrus | L | -54 | -22 | -22 | 1.70 |
| OFC | L | -48 | 20 | -8 | 1.82 |
| OFC | R | 50 | 22 | -8 | 1.64 |
| **SDN – IDN** |  |  |  |  |  |
| Precentral gyrus | R | 44 | 0 | 50 | 1.65 |
| Superior parietal lobule | R | 44 | -44 | 60 |  |
| Insula | R | 34 | 16 | 8 | 1.63 |
| Middle frontal gyrus | R | 34 | 2 | 62 | 1.63 |
| **SDN – SC** |  |  |  |  |  |
| Superior frontal gyrus | R | 8 | 44 | 40 | 1.63 |
| Frontal pole | L | -40 | 44 | -10 | 1.89 |
| Frontal pole | R | 32 | 44 | 10 | 2.44 |
| OFC | L | -32 | 22 | 10 | 2.43 |
| OFC | R | 22 | 22 | -12 |  |
| Paracingulate gyrus | L | -8 | 22 | 44 | 2.09 |
| Superior frontal gyrus | R | 6 | 46 | 42 | 1.92 |
| Pallidum | R | 14 | 0 | -6 | 1.97 |
| Insula | L | -32 | 20 | -8 | 2.51 |
| Putamen | R | 20 | 22 | -6 | 1.92 |
| **SC – SDN** |  |  |  |  |  |
| Paracingulate | L | 0 | 22 | 44 | 2.61 |
| Paracingulate | R | 6 | -44 | -4 | 1.77 |
| Anterior cingulate gyrus | R | 6 | 14 | 20 | 1.78 |
| Posterior cingulate gyrus | L | 0 | -38 | 26 | 2.64 |
| Precuneus | L | 0 | -62 | 40 | 2.12` |
| Precuneus | R | 4 | -60 | 50 | 1.94 |
| Middle frontal gyrus | R | 40 | 32 | 26 | 2.56 |
| Middle frontal gyrus | L | -38 | 32 | 26 | 2.03 |
| Inferior frontal gyrus | L | -48 | 14 | 26 | 2.36 |
| Inferior frontal gyrus | R | 50 | 20 | 26 | 2.04 |
| Frontal pole | R | 50 | 38 | -4 | 1.63 |
| Supramarginal gyrus | L | -38 | -36 | 38 | 2.10 |
| **SC – IDN** |  |  |  |  |  |
| Middle frontal gyrus | L | -32 | 36 | 26 | 1.60 |
| Putamen | L | -32 | 10 | -2 | 1.58 |
| Precentral gyrus | L | -12 | -34 | 46 | 3.18 |
| Precuneus | L | 0 | -58 | 38 | 2.51 |
| Anterior cingulate gyrus | R | 0 | 36 | -4 | 1.98 |
| Insula | R | 36 | 20 | -2 | 1.88 |
| Caudate | L | -14 | 16 | 0 | 1.82 |
